# Endoscopic Deflux Injection for Vesicoureteral Reflux in a Renal Allograft Complicated by Recurrent Acute Pyelonephritis: A Case Report

**DOI:** 10.7759/cureus.98927

**Published:** 2025-12-10

**Authors:** Tomohiro Matsuo, Tsuyoshi Matsuda, Kensuke Mitsunari, Kojiro Ohba, Ryoichi Imamura

**Affiliations:** 1 Department of Urology, Graduate School of Biomedical Sciences, Nagasaki University, Nagasaki, JPN

**Keywords:** acute pyelonephritis, deflux injection, kidney transplantation, renal allograft, vesicoureteral reflux

## Abstract

Vesicoureteral reflux (VUR) in a renal allograft ureter is a well-recognized and clinically significant complication that may lead to recurrent febrile urinary tract infection (fUTI) and progressive allograft dysfunction. While open ureteral reimplantation remains a standard surgical option, it is relatively invasive for kidney transplant recipients. Endoscopic subureteral injection of a bulking agent such as Deflux has been widely used for primary VUR in children, but reports in renal transplant recipients are limited.

We report a case of a living donor kidney transplant recipient who developed recurrent episodes of acute pyelonephritis in the transplanted kidney. Imaging studies revealed clinically significant VUR at the ureteroneocystostomy site of the renal allograft. Despite prophylactic antibiotics, febrile urinary tract infections persisted, raising concern for long-term graft injury. The patient underwent endoscopic anti-reflux surgery using subureteral Deflux injection under cystoscopic guidance. The procedure was completed without intraoperative or perioperative complications. Follow-up voiding cystography demonstrated resolution of VUR, and no further episodes of acute pyelonephritis occurred during 52 months of clinical follow-up, with stable allograft function.

This case suggests that endoscopic Deflux injection can be a safe and effective minimally invasive option for managing transplant kidney VUR complicated by recurrent acute pyelonephritis, potentially preserving renal allograft function while avoiding more invasive reconstructive surgery.

## Introduction

Kidney transplantation is the treatment of choice for patients with end-stage renal disease, but urological complications still have an important impact on quality of life and long-term graft outcomes. Vesicoureteral reflux (VUR), defined as the backward flow of urine from the bladder into the ureter and renal pelvis, is relatively common in renal allografts, with reported incidences ranging from low to very high depending on recipient background, bladder function, and the ureteroneocystostomy technique used. Recent reviews have highlighted that dialysis vintage and reduced bladder capacity are key recipient-related risk factors for post-transplant VUR and that lower urinary tract dysfunction, including dysfunctional voiding, may also contribute to its development, although its precise impact on graft survival remains a matter of debate, particularly in the absence of recurrent infection [1]. In pediatric transplant recipients, an online survey by the European Society for Paediatric Nephrology confirmed that allograft VUR is a frequent finding and that diagnostic and therapeutic strategies vary considerably among centers, reflecting the lack of standardized management [2].

Management of VUR in kidney transplant recipients is usually individualized according to reflux grade, symptom burden, and allograft function. Conservative approaches, including antibiotic prophylaxis and optimization of bladder function, may be considered for low-grade or mildly symptomatic post-transplant VUR, whereas patients with high-grade reflux or recurrent febrile urinary tract infections (fUTIs; UTIs accompanied by fever and systemic signs of infection) are often managed with surgical or endoscopic intervention [1,2]. Open or minimally invasive ureteral reimplantation has long been regarded as a reference standard, but it can be technically challenging in immunosuppressed recipients with prior pelvic surgery or a hostile bladder and is associated with non-negligible perioperative morbidity. In this context, endoscopic subureteral injection of bulking agents has emerged as a minimally invasive, graft-preserving alternative for symptomatic post-transplant VUR [3,4].

Deflux, a dextranomer/hyaluronic acid copolymer, is an established bulking agent for endoscopic treatment of primary VUR in children and has more recently been applied to VUR involving renal allografts [3-5]. A recent systematic review and several small adult and pediatric series suggest that subureteral Deflux injection in kidney transplant recipients is technically feasible and generally safe, but overall success rates are modest, most data originate from small retrospective cohorts, and there is a non-negligible risk of ureterovesical junction obstruction [3-5]. These reports emphasize the need for prospective studies to clarify optimal indications and long-term outcomes.

However, detailed technical descriptions and long-term outcomes in adult living donor recipients with clinically significant but low-grade VUR complicated by recurrent acute pyelonephritis remain limited. Here, we report a young adult living donor kidney transplant recipient with grade II allograft VUR, recurrent acute pyelonephritis, and transient graft dysfunction who was successfully managed with double-hydrodistention implantation technique (HIT)-based Deflux injection and ureteral stent-assisted alignment, highlighting a minimally invasive option for clinically significant allograft VUR in this setting.

## Case presentation

A 35-year-old woman with end-stage renal disease secondary to IgA nephropathy underwent a living donor kidney transplantation at 33 years of age, with her mother serving as the donor. Her bilateral native kidneys had not been removed and remained in situ. The donor’s left kidney was implanted in the recipient’s right iliac fossa, with end-to-side anastomoses of the renal artery and vein to the external iliac artery and vein. An extravesical ureteroneocystostomy was performed according to the Lich-Gregoir technique with creation of a submucosal tunnel to provide an anti-reflux mechanism. The early postoperative course was uneventful, and graft function stabilized with a serum creatinine level of approximately 1.2 mg/dL (reference range for adult women in our laboratory: 0.46-0.79 mg/dL).

At one year and one month after transplantation, she developed her first episode of fUTI consistent with acute pyelonephritis of the transplanted kidney, which resolved with intravenous followed by oral antibiotics. Thereafter, she experienced multiple recurrent episodes of acute pyelonephritis despite the introduction of prophylactic antibiotics. During this period, repeated fUTIs and infection-related dehydration led to a transient deterioration of graft function, with the serum creatinine level rising from a baseline of approximately 1.2 mg/dL to a maximum of 3.30 mg/dL. This pattern of recurrent febrile infection and transient graft dysfunction prompted further evaluation for an anatomic cause.

**Figure 1:**
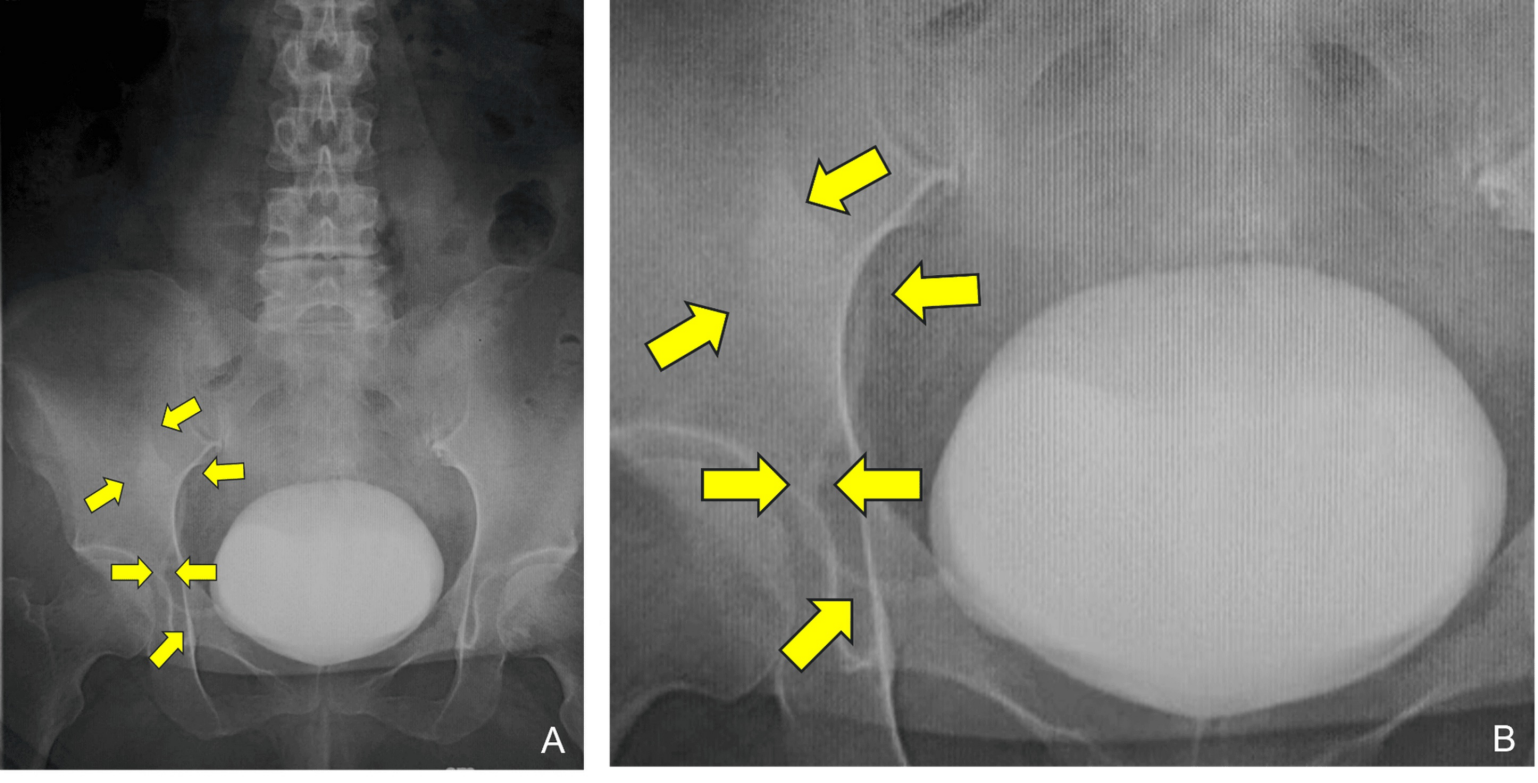
Preoperative voiding cystourethrography of the renal allograft (A) Full bladder view during the filling phase, showing the transplant ureteral orifice on the right anterior bladder wall. (B) Magnified view of the region around the transplant ureteral orifice shown in (A), demonstrating contrast material refluxing from the transplant ureteral orifice into the transplant ureter and collecting system of the renal allograft, consistent with grade II vesicoureteral reflux (arrow: refluxed contrast within the transplant ureter).

Voiding cystourethrography demonstrated grade II VUR, according to the International Reflux Study Committee grading system, from the bladder into the ureter and collecting system of the transplanted kidney on the graft side (Figure 1A, 1B).

Although this reflux was classified as grade II, which is generally regarded as low-grade, it was considered clinically significant in light of the associated recurrent febrile pyelonephritis and transient allograft dysfunction; therefore, active correction was planned. Treatment options, including continued conservative management with prophylactic antibiotics and surgical or endoscopic correction, were discussed with the patient, and she elected to undergo endoscopic anti-reflux surgery.

Two years and six months after kidney transplantation, she was admitted for endoscopic correction of vesicoureteral reflux. Under spinal anesthesia, cystoscopy was performed, and the transplant ureteral orifice was identified on the anterior bladder wall, slightly to the right side. The ureteral orifice appeared mildly dilated. A ureteral stent was temporarily inserted to adjust the position and alignment of the intramural portion of the transplant ureter, thereby facilitating submucosal injection using a metallic injection needle. Subsequently, a dextranomer/hyaluronic acid copolymer (Deflux) was injected submucosally beneath the transplanted ureteral orifice according to the double-HIT technique (Video 1) [6], with a total volume of 2 mL injected.

**Video 1:** Endoscopic subureteric Deflux injection for vesicoureteral reflux

A satisfactory mound was created, and the ureteral orifice assumed a slit-like configuration at the end of the procedure. No intraoperative complications were observed. The urethral catheter was removed on the first postoperative day, and the patient was discharged on postoperative day 2.

**Figure 2:**
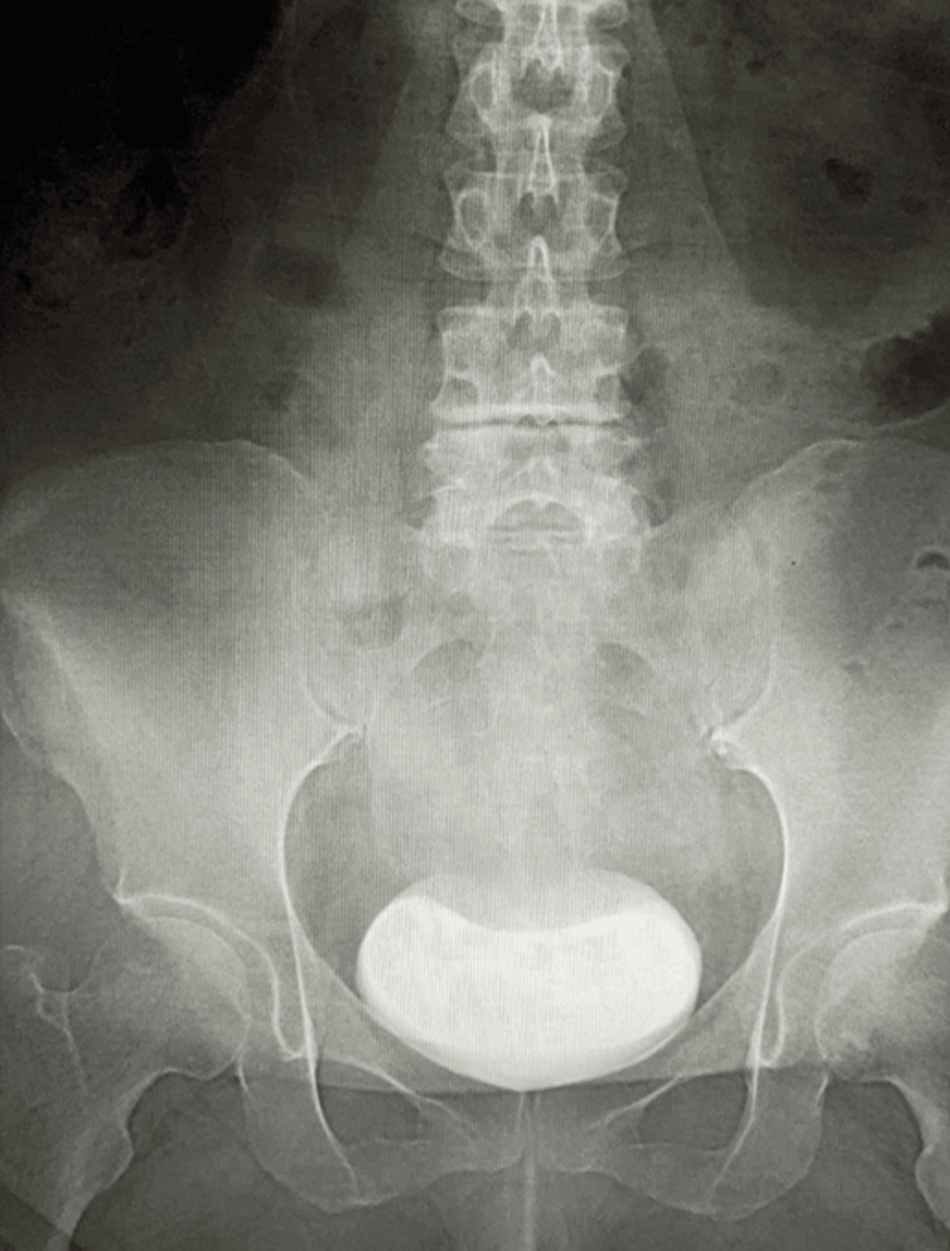
Voiding cystourethrography three months after Deflux injection Follow-up imaging performed three months after endoscopic Deflux injection shows no contrast reflux into the transplant ureter or collecting system of the renal allograft, indicating complete radiologic resolution of vesicoureteral reflux.

The postoperative course was uneventful. A follow-up voiding cystourethrogram performed three months after the endoscopic procedure showed complete resolution of vesicoureteral reflux into the transplanted kidney (Figure 2).

Four years and four months after Deflux injection, no further episodes of acute pyelonephritis or fUTI had occurred, and serial urinalysis and urine cultures remained negative without evidence of asymptomatic bacteriuria. Her serum creatinine level had improved to 1.18 mg/dL, returning to approximately the pre-infection baseline, and graft function remained stable without evidence of procedure-related complications such as ureteral obstruction.

## Discussion

VUR of the renal allograft is a relatively frequent urological complication after kidney transplantation, although reported incidences vary widely depending on study design, patient selection, and diagnostic strategy. A recent systematic review highlighted this variability and identified dialysis vintage and atrophic bladder as key recipient-related determinants, while the role of ureteral implantation technique remains controversial [1]. Molenaar et al. reviewed adult kidney transplant cohorts and noted that post-transplant VUR is reported in roughly 10% of recipients in some series but emphasized that its clinical impact on graft function and survival remains uncertain, particularly in the absence of recurrent infection [7]. In children and young people, Hewitt et al. similarly showed that VUR into transplanted kidneys is common in pediatric recipients [8]. Our patient developed clinically significant VUR of the transplant ureter relatively early, at one year and one month after living donor kidney transplantation, in the setting of a presumably small, previously uremic bladder.

fUTIs are among the most common infectious complications after renal transplantation and can lead to both short-term and long-term morbidity. John et al. demonstrated a high prevalence of fUTI after pediatric renal transplantation, with episodes associated with hospitalization and transient graft dysfunction [9]. Erturk et al. specifically evaluated patients with VUR after renal transplantation and reported that 56% experienced UTIs and that three-year graft survival was only 50%, suggesting that VUR associated with infection may adversely affect graft outcomes [10]. In contrast, some studies have suggested that asymptomatic, low-grade VUR without recurrent infection may not independently worsen graft survival, underscoring the need to individualize management based on infection burden rather than reflux grade alone [7]. In our case, the patient suffered multiple episodes of acute pyelonephritis despite prophylactic antibiotics, and her serum creatinine worsened transiently from a stable baseline of approximately 1.2 mg/dL to 3.30 mg/dL, indicating clinically relevant allograft injury rather than “benign” reflux.

Management options for post-transplant VUR include conservative treatment with antibiotic prophylaxis, endoscopic injection of bulking agents, and open or laparoscopic ureteral reimplantation [1,7]. Although prophylactic antibiotics may reduce urinary tract infection episodes, they do not correct the underlying reflux, and high-grade VUR or VUR associated with recurrent fUTIs generally warrants active intervention. In our case, despite the reflux being classified as grade II, the combination of recurrent febrile pyelonephritis and transient allograft dysfunction was considered sufficient to justify active correction of VUR rather than continued conservative management. Hirose et al. reported that treatment of VUR after kidney transplantation, mainly by surgical correction, was associated with prevention of graft function deterioration and enabled long-term graft survival [11]. Varaschin et al. similarly showed that surgical correction of post-transplant VUR significantly reduced fUTI episodes in a contemporary adult cohort [12]. In our young living donor recipient, prophylactic antibiotics failed to prevent recurrent pyelonephritis, and the risk of cumulative infection-related damage to an otherwise good graft was considered unacceptable; therefore, definitive correction of VUR was deemed necessary.

Open ureteral reimplantation has long been regarded as the reference standard for surgically managing post-transplant VUR, with high rates of radiologic resolution and reduction in UTI burden [11,12]. However, ureteral reimplantation in a transplanted ureter is often technically demanding because prior surgery and altered pelvic anatomy can limit ureteral mobility and vascularity, and it may be associated with greater perioperative morbidity and longer hospitalization compared with minimally invasive approaches. For a relatively young, otherwise healthy living donor recipient such as ours, a minimally invasive option that could control infection while avoiding reoperative pelvic surgery was therefore preferred.

Endoscopic subureteral injection of bulking agents has emerged as a graft-preserving alternative for symptomatic post-transplant VUR. Several series have shown that endoscopic Deflux injection for post-transplant VUR is safe and feasible, achieving clinical or radiologic success in approximately half to three-quarters of patients, with low complication rates [3,13,14]. Taken together, these studies support endoscopic Deflux injection as an intermediate option between conservative management and open reimplantation, offering reasonable success with lower morbidity.

Technical refinements have also contributed to improved outcomes of Deflux injection. Kirsch and Arlen described the intraureteric hydrodistention implantation technique (HIT) and the double-HIT technique, which create intraureteric implants and were associated with higher radiologic success rates compared with the original subureteral transurethral injection (STING) method in primary VUR [6]. Although these data derive mainly from pediatric patients with native kidneys, the underlying concepts, improving tunnel coaptation and creating a more proximal intraureteric mound, are highly relevant to transplant ureters, which often have short tunnels and patulous orifices. In our patient, we applied a double-HIT-based Deflux injection at the transplant ureteral orifice after temporarily inserting a ureteral stent to optimize the alignment of the intramural ureter and facilitate accurate submucosal injection. This resulted in a robust mound, a slit-like configuration of the ureteral orifice, and complete radiologic resolution of VUR at three months, with no recurrence of acute pyelonephritis over four years and four months of follow-up and recovery of serum creatinine to 1.18 mg/dL. Given the often shorter and anatomically constrained nature of the transplant ureter, the use of this double-HIT-based technique, aimed at achieving a more reliable valve mechanism than the simple STING method, combined with ureteral stent-assisted alignment, highly likely contributed to the long-term success observed in this case.

Ureteral obstruction is a recognized but relatively uncommon complication after Deflux injection. Although it has been reported only rarely in large pediatric series of endoscopic Deflux treatment for primary VUR, concern remains that the shorter and more anatomically constrained transplant ureter may be at relatively higher risk of obstruction. A recent case report by Aoki et al. described acute kidney injury due to ureteral obstruction immediately after Deflux injection for VUR in a pediatric kidney transplant recipient, highlighting that bulking agents can precipitate clinically significant obstruction in this setting [15]. In our adult living donor recipient, no obstruction or hydronephrosis was observed during long-term follow-up, but this potential risk underscores the need for careful patient selection, meticulous injection technique, and close postoperative surveillance, particularly in recipients with impaired bladder function or high-grade VUR.

Over 52 months of follow-up after Deflux injection, our patient experienced no further episodes of acute pyelonephritis and maintained serum creatinine at approximately 1.2 mg/dL. Compared with previously published series, the present case illustrates several clinically relevant points. First, the patient was a relatively young adult with a living donor renal allograft and recurrent febrile pyelonephritis despite prophylactic antibiotics, representing a scenario in which both infection control and graft preservation are crucial. Second, Deflux injection using a double-HIT-based technique achieved durable elimination of VUR and infections without the need for open reimplantation, thus controlling symptoms while avoiding the additional morbidity and vascular risk associated with repeat pelvic surgery. Third, normalization of serum creatinine over long-term follow-up suggests that timely correction of symptomatic VUR may not only reduce infection burden but also prevent progressive allograft dysfunction, consistent with recent data supporting active treatment of clinically significant VUR after transplantation [11,12]. While a single case report cannot define treatment algorithms, our experience adds to the growing evidence that endoscopic Deflux injection is a valuable, kidney-sparing option for symptomatic allograft VUR in carefully selected patients.

## Conclusions

In this single case of a young adult living donor kidney transplant recipient, even low-grade vesicoureteral reflux in a renal allograft was clinically significant when associated with recurrent febrile urinary tract infections and transient allograft dysfunction. Endoscopic Deflux injection using a double-HIT-based technique achieved durable resolution of reflux, freedom from acute pyelonephritis, and recovery of graft function without procedure-related obstruction. These observations suggest that Deflux injection may represent a minimally invasive, graft-sparing option for carefully selected kidney transplant recipients with symptomatic VUR in whom conservative therapy has failed, but further data are needed before firm treatment recommendations can be made.
